# Clinical utility of circulating tumor-associated cells to predict and monitor chemo-response in solid tumors

**DOI:** 10.1007/s00280-020-04189-8

**Published:** 2020-11-10

**Authors:** Timothy Crook, Andrew Gaya, Raymond Page, Sewanti Limaye, Anantbhushan Ranade, Amit Bhatt, Sanket Patil, Prashant Kumar, Darshana Patil, Dadasaheb Akolkar

**Affiliations:** 1 Department of Oncology, Broomsfield Hospital, Chelmsford, UK; 2 HCA Healthcare UK, London, W1G 6AF UK; 3 Worcester Polytechnic Institute, Worcester, USA; 4 Department of Medical Oncology, Kokilaben Dhirubai Ambani Hospital, Mumbai, India; 5 Department of Medical Oncology, Avinash Cancer Clinic, Pune, India; 6 Datar Cancer Genetics, F-8 D-Road, Ambad, Nasik, 422010 India; 7 Institute of Bioinformatics, International Technology Park, Bangalore, 560066 Karnataka India; 8 Manipal Academy of Higher Education (MAHE), Manipal, 576104 Karnataka India

**Keywords:** Circulating tumor-associated cells: C-TACs, In vitro chemoresponse profiling: CRP, Non-invasive liquid biopsy, Surveillance, Precision oncology, Chemotherapy

## Abstract

**Purpose:**

Selection of cytotoxic chemotherapy agents (CCA) based on pre-treatment evaluation of drug sensitivities is a desirable but unmet goal for personalized anticancer treatment strategies. Prior attempts to correlate *in* vitro Chemo-Response Profiles (CRP) of tumor explants or Circulating Tumor Cells (CTCs) with clinical outcomes have been largely unsuccessful.

**Methods:**

We present results from a large cohort (*n* = 5090, three Arms) of patients with various solid organ tumors, where CRP of Circulating Tumor-Associated Cells (C-TACs) was determined against cancer-specific CCA panels to generate a database of 56,466 unique CRP.

**Results:**

In Arm 1 (*n* = 230), 93.7% concordance was observed between CRP of C-TACs and concurrently obtained Tumor tissue Derived Cells (TDCs). In arm 2 (*n* = 2201, pretreated), resistance of C-TACs to ≥ 1 CCA was observed in 79% of cases. In a blinded subset analysis of 143 pretreated patients with radiologically ascertained disease progression, CRP of C-TACs was 87% concordant with in vivo treatment failure. In Arm 3 (*n* = 2734, therapy naïve), innate resistance of C-TACs to ≥ 1 CCA was observed in 61% of cases. In a blinded subset analysis of 77 therapy naïve patients, in vitro chemo-sensitivity of C-TACs was concordant with radiologically ascertained treatment response to first line CCA in 97% of cases.

**Conclusion:**

To our knowledge, this is the first expansive and in-depth study demonstrating that real-time CRP of C-TACs is a viable approach for non-invasive assessment of response to CCA in solid organ cancers.

**Electronic supplementary material:**

The online version of this article (10.1007/s00280-020-04189-8) contains supplementary material, which is available to authorized users.

## Introduction

Despite the development of targeted anticancer therapies such as Tyrosine Kinase Inhibitors (TKI) and Immune Checkpoint Inhibitors (ICI), Cytotoxic Chemotherapy Agents (CCA) remain essential agents in the neo-adjuvant, adjuvant and metastatic settings in the management of most solid tumors. Choice of monotherapy or combination chemotherapy regimens is largely based on clinical guidelines with minimal or no guidance from molecular or functional indications. This inability to inform optimal therapy in individual patients and subsequently low response rates reflect the limitations of such non-personalized therapy selection. For example, in metastatic breast cancer, first-line therapy with Standard of Care (SoC) weekly Taxol typically produces an overall response rate of ~ 30% with a further ~ 30% of patients achieving stable disease [1], implying that 40% of patients will derive no benefit at all but will incur toxicity.

The failure of chemotherapy can be attributed to resistance of tumors (innate and acquired) towards CCA and is a significant impediment to successful management of solid tumors [2, 3]. Resistance to CCA is random, and hence unpredictable, and becomes apparent only at response evaluation imaging or clinical assessment. This inability to detect emerging sub-clinical drug resistance in real time is the undeniable Achilles heel of purposive strategic vigilance against treatment failure.

Understanding the resistance/sensitivity profile of each patient’s case prior to initiation of treatment offers the ability to optimize treatments and time-dependent clinical outcomes not only at first but at all subsequent lines of therapy. Prior attempts at in vitro chemoresistance profiling (CRP) of tumor tissue-derived cells (TDCs) showed inadequate clinical correlation and hence is not widely adopted in clinical practice [4, 5]. There have been prior attempts [6–10] to develop real-time non-invasive means to monitor cancer sensitivity to CCA based on circulating tumor cells (CTCs), but these generally have suffered from low yields of CTC which hinders any meaningful clinical application of the concept. The scope of CTC investigations has been largely limited to enumeration for the purposes of prognostication [11].

We have recently described a method that permits detection, enrichment and harvest of viable circulating tumor-associated cells (C-TACs: EpCAM + , Pan-CK + , CD45 ±) from the peripheral blood of patients with various solid organ cancers [12]. We employed this approach to enrich and harvest C-TACs from 5,090 patients with prior diagnosis of either of 17 types of solid organ cancers, irrespective of treatment status and extent of disease. In a subset of 230 patients, viable TDCs were harvested from concurrently biopsied tumor tissue. In vitro response profiling of C-TACs against a panel of CCA was performed to determine concordance in response with TDCs, concordance with radiological treatment response, and to identify innate and acquired resistance in therapy naïve and pretreated cases. We present findings that establish CRP of C-TACs as an accurate and patient friendly means to non-invasively monitor resistance to CCA and guide selection of optimal treatments.

## Methods

### Study design

The present manuscript reports findings of exploratory investigations from three prospective interventional trials and one prospective observational trial. The interventional trials are: (a) “*The assessment of potential benefits of molecular analysis and *in vitro* chemo response directed at opening treatment options for relapsed and refractory metastatic solid organ tumors.—RESILIeNT*” [13] (WHO ICTRP ID CTRI/2018/02/011808), (b) “*A two arm randomized open label prospective parallel design superiority Phase II clinical trial to evaluate the efficacy of a therapy administered based on guidance obtained from integrative molecular and in-vitro chemosensitivity analysis provided by the DCGL investigation platform (Exacta™) versus standard of care therapy in newly diagnosed therapy naïve advanced/unresectable gallbladder cancer, cholangiocarcinoma, pancreatic cancer, hepatocellular carcinoma, gastric cancer, esophageal cancer and glioblastoma.—ACTPrO*” (WHO ICTRP ID CTRI/2018/05/014,178), (c) “*To evaluate the efficacy of therapy administered based on guidance obtained from integrative molecular analysis of cell free nucleic acids and *in vitro* chemosensitivity analysis of circulating tumor cells, aimed at improving availability of therapy options and treatment outcomes in relapsed/refractory metastatic solid organ tumors with unavailability of *de novo* tissue biopsies.—LIQUID-IMPACT*” (WHO ICTRP ID CTRI/2019/02/017,548). The observational study is “*Tissue biopsy Replacement with Unique Evaluation of circulating bio-markers for morphological evaluation and clinically relevant molecular typing of malignancies from BLOOD sample—TrueBlood*” (WHO ICTRP ID CTRI/2019/03/017918).

This manuscript does not report the primary study outcomes or the primary efficacy endpoints for any of the above trials. Study outcomes for the RESILIENT Trial have already been published [13], while those for ACTPrO, LIQUID-IMPACT and TrueBlood trials will be published separately. All studies were approved by the Institutional Ethics Committee of the study sponsor Datar Cancer Genetics (DCG) as well as other participating centers. Details of all studies are available at WHO ICTRP (https://apps.who.int/trialsearch/Default.aspx) and can be accessed using the study IDs given above. All studies were conducted in accordance with existing ethical guidelines such as the International Council for Harmonization of Technical Requirements for Pharmaceuticals for Human Use (ICH) as well as the Declaration of Helsinki.

### Study population

**Fig. 1:**
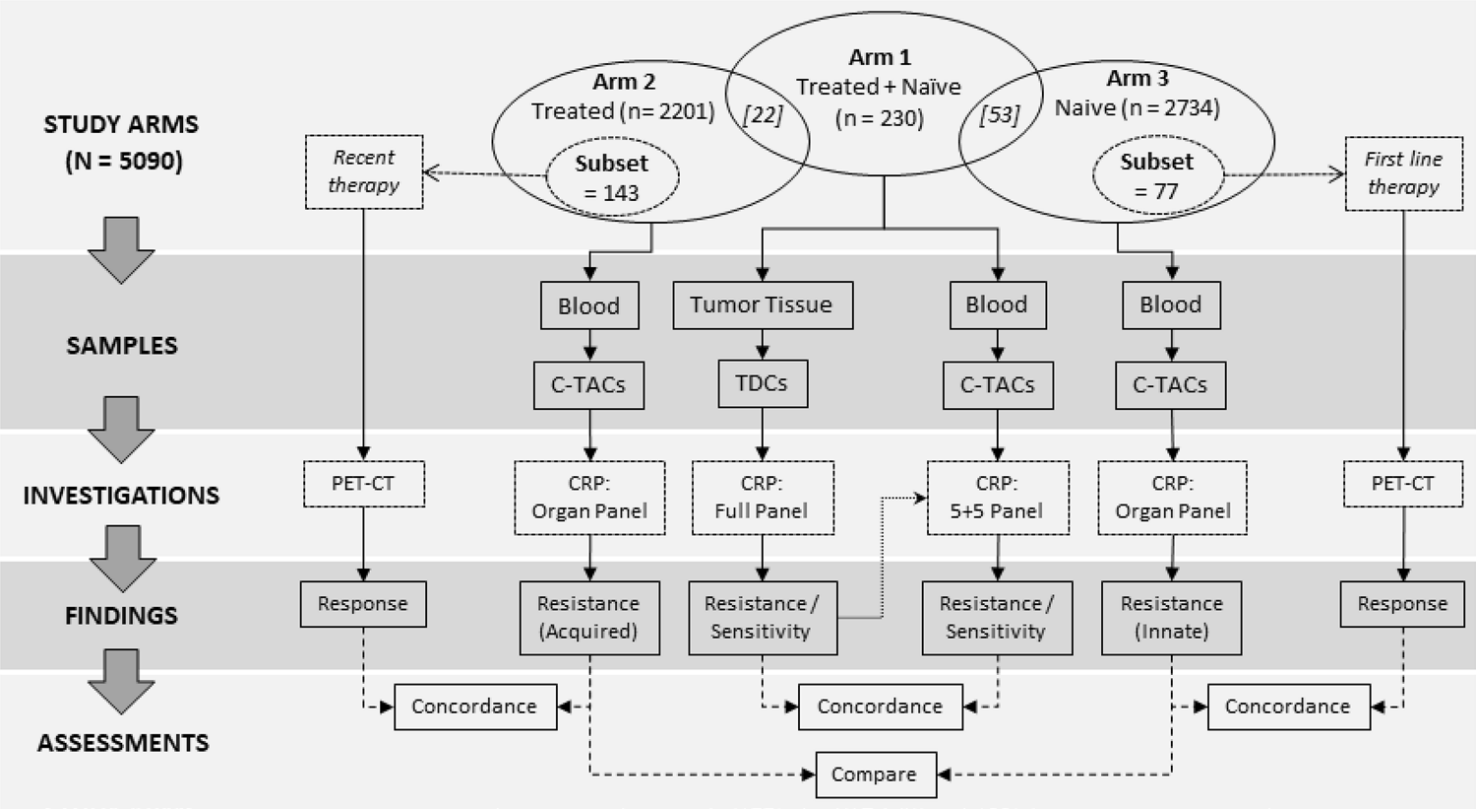
Study design. CRP of C-TACs in previously diagnosed cases (*n* = 5090) of cancers. Arm 1 evaluated CRP concordance between C-TACs and concurrently obtained TDCs. Arm 2 evaluated acquired chemoresistance based on prior treatments. Arm 3 evaluated innate chemoresistance in therapy naïve patients. Patients overlapping between Arm 1 and Arm 2 and between Arm 1 and Arm 3, respectively, are indicated in italics in the Venn Diagram within square parentheses

At the time of enrollment, all eligible volunteers were counselled regarding the study procedures as well as primary, secondary and exploratory aims of respective studies. Eligible and willing participants who provided signed informed consent were enrolled into each study. For the purpose of the present manuscript, patients in the study cohort (*n* = 5090) were retrospectively assigned to one of three main study arms (Fig. 1) depending on therapy status and availability of biopsied tumor tissue. Arm 1 comprised 230 patients (therapy naïve as well as previously treated with CCA) from whom peripheral blood and freshly biopsied tumor tissue was obtained. Arm 2 comprised 2201 patients who had previously received CCA and from whom blood was collected. Arm 3 included 2734 therapy naïve patients from whom blood was collected. 22 patients who had previously received CCA were common to Arm 1 and Arm 2, while 53 recently diagnosed and therapy naïve patients were common to Arm 1 and Arm 3. Patient demographics are provided in Table 1.

**Table 1:** Patient demographics

Parameter	Arm 1	Arm 2	Arm 3	Overall
Gender				
Male	81	869	1275	2203
Female	140	1332	1459	2887
Total	230	2201	2734	5090
Age (years)				
Median	53	54	57	55
Range	(18–88)	(16–85)	(15–95)	(15–95)
Therapy status				
Treated	64	2201	0	2243
Therapy Naïve	166	0	2734	2847
Disease status				
Metastatic	104	1648	1840	3557
Non-metastatic	69	399	289	740
Unavailable	57	154	605	793
Cancer type				
Bladder	–	47	26	73
Breast	68	681	685	1410
Cervix	19	139	159	308
Colorectum	20	205	173	393
Gallbladder	2	30	45	75
Head and neck	71	485	525	1064
Lung	8	168	320	492
Neuroendocrine	–	13	11	24
Oesophagus	7	89	140	235
Ovary	16	147	78	232
Pancreas	3	50	63	116
Prostate	5	3	113	120
Stomach	1	45	69	115
Testes	–	16	17	33
Thyroid	3	18	40	60
Unknown Primary	–	22	218	240
Uterus	7	43	52	100

Chemoresistance of C-TACs was profiled from 5090 patients across the three Study Arms

### Tissue and blood samples

Peripheral blood (15 mL) was collected in EDTA vacutainer tubes from all (*n* = 5090) study participants. For patients in Arm 1, blood was collected prior to biopsy for obtaining paired fresh tumor tissue. All samples were processed and assays conducted at the facilities of the Study Sponsor, which offers CAP and CLIA accredited services and is also accredited for ISO 15189:2012 compliance by the National Accreditation Board for Testing and Calibration Laboratories (NABL), which is the International Laboratory Accreditation Cooperation (ILAC) Agency for India.

### Harvest of circulating tumor-associated cells (C-TACs)

C-TACs were enriched and harvested from Peripheral Blood Mononuclear Cells (PBMCs) as described previously [10]. Briefly, PBMCs were treated with epigenetically activating media for up to 100 h at 37 °C under 5% CO_2_, 4% O_2_. This process induces cell death in normal (non-malignant) cells with functional apoptotic machinery while simultaneously conferring survival privilege on apoptosis-resistant cells of tumorigenic origin, i.e. circulating tumor-associated cells (C-TACs) and their heterotypic clusters (C-ETACs: circulating ensembles of tumor-associated cells). C-TACs (EpCAM + , PanCK + , CD45 ±) include CTCs (EpCAM + , PanCK + , CD45-) as well as other cell types such as tumor-associated macrophages (TAMs) and tumor-associated fibroblasts (TAFs). Supplementary Table S1 provides the C-TAC yields in various cancer types and based on treatment status. Enriched and harvested C-TACs were identified by immunocytochemistry (ICC) profiling for EpCAM, Pan-CK and CD45, as well as Organ and Subtype-Specific (OSS) markers to verify cancer type (Supplementary Figure S1, Supplementary Figure S2, Supplementary Table S2). Fluorescence imaging was performed on Cell Insight CX7 High-Content Screening Platform (ThermoFisher Scientific, USA).

### Harvesting of viable TDCs

Tumor tissue (Arm 1) was evaluated for tumor content (minimum requirement > 70%) by histopathological analysis by an experienced pathologist. Tumor tissues were dissociated into single-cell suspensions by a combination of mechanical dissociation and enzymatic degradation of the extracellular matrix using the Tumor Cell Isolation Kit, human kit components and the gentleMACS™ Dissociator (Miltenyi Biotech, Germany). The single cell suspension of tumor-derived cells (TDCs) obtained by this method was cultured at 37 °C under 5% CO_2_ and 4% O_2_ for 24 h and viable TDCs were then harvested for further applications such as CRP.

### In vitro chemotherapy sensitivity analysis

The in vitro chemosensitivity assay was designed to evaluate the sensitivity of viable TDCs, CTACs or cell lines to various chemotherapeutic agents. The test concentration for each CCA was based on reported peak plasma concentration at the recommended clinical dose. The cytotoxicity of CCA were preliminarily evaluated on SKBR3 (ATCC^®^ HTB-30™), SW620 (ATCC^®^ CCL-227™) and RCC 769-P (ATCC^®^ CRL-1933™) cell lines (Supplementary Table S3) and then on primary TDCs. Approximately, 10^4^ cells/well were seeded into 96-well culture plates and treated with CCAs at 37 °C, for 30 min, under 5% CO_2_ and 4% O_2_. The plates were transferred into the incubator chamber of a microplate reader (VarioScan LUX, Thermo Fisher Scientific) where absorbance (*λ* = 600 nm) was recorded every 5 min over 12 h. Change in absorbance which correlates with apoptosis was converted to kinetic units (KU) of apoptosis as described previously [14]. Baseline apoptotic events were accounted for by using control wells with untreated cells. Active apoptosis was indicated as > 1.0 KU. The five most active (highest cell death) and five least active drugs (lowest or no cell death) were identified for each TDC sample to generate the ‘5 + 5 Drug Panel’.

### CCA cytotoxicity analysis of C-TACs

**Fig. 2:**
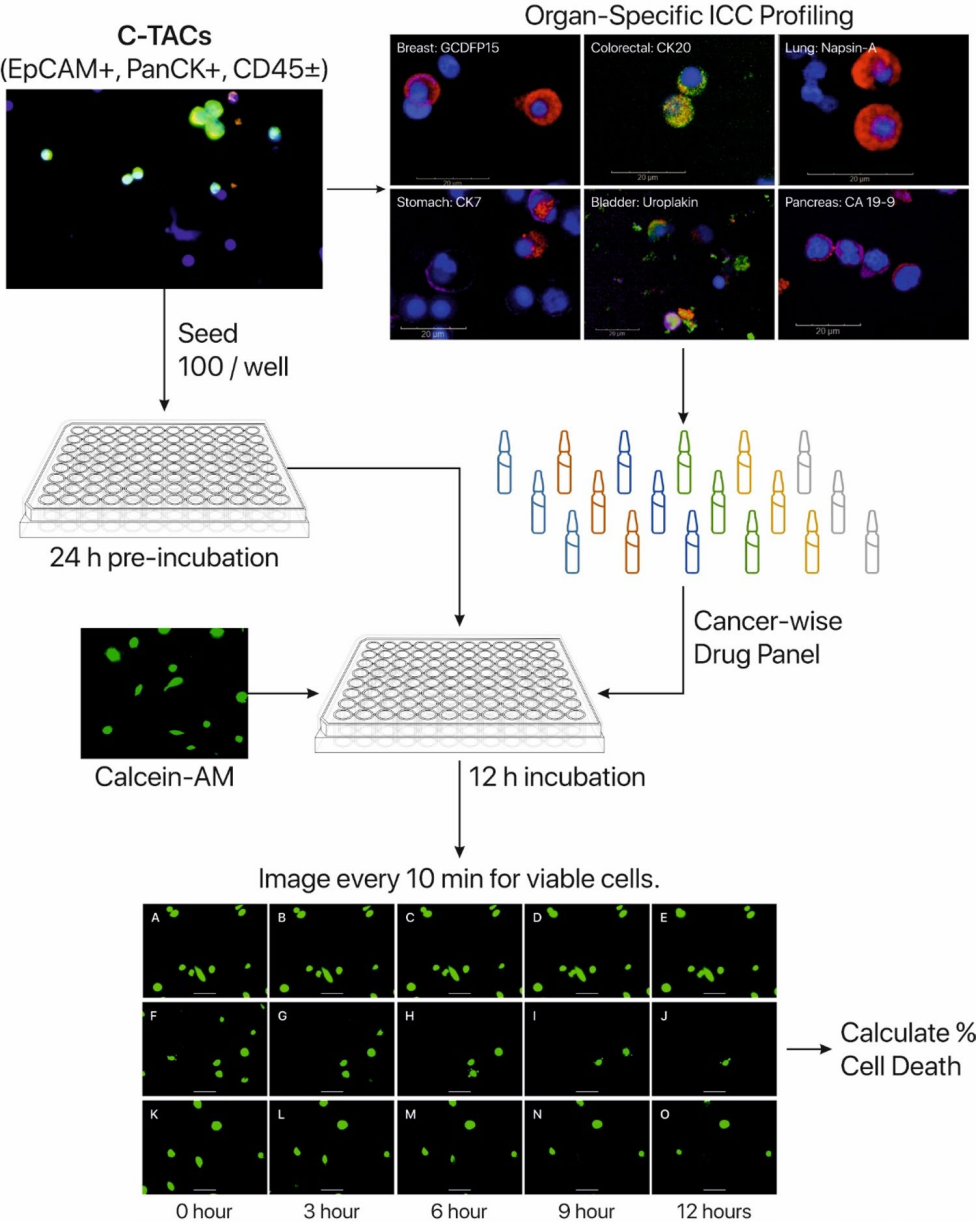
In vitro CRP workflow. C-TACs were ascertained by ICC profiling with OSS markers to identify cancer-specific drug panel. C-TACs were seeded into multi-well assay plates, pre-incubated and treated with appropriate CCA panel. C-TACs are stained with Calcein-AM to monitor viable cells during time-lapse fluorescent imaging where images were obtained every 10 min for 12 h. Proportion of surviving C-TACs were estimated to determine % cell death. Panels A-O show representative images of surviving C-TACs at various time points, when treated with different drugs with either low/no cytotoxicity (**a–e**), high cytotoxicity (**f–j**) and moderate cytotoxicity (**k–o**). Also see Supplementary video

Approximately, 100 C-TACs/well were seeded into 96-well culture plates and incubated for 24 h (37 °C, 5% CO_2_, 4% O_2_). Viable cells were stained with Calcein AM and treated with optimized concentrations of CCAs. Each plate included control wells (no drug) to determine baseline mortality as well as positive (known cytotoxicity) controls with SKBR3, SW620 or RCC 769-P cells. The plates were placed in the on-stage incubator of fluorescent microscope EVOS M7000 (Thermo Fisher Scientific) at 37 °C with 5% CO_2_, 4% O_2_ and the wells imaged every 10 min for 12 h (Supplementary Video). Figure 2 depicts the CRP scheme. Extent of cell death was determined based on cell morphology changes and time required for fade out of live cell tracking dye. For Arm 1 samples, C-TACs were treated with the ‘5 + 5 Drug Panel’ to determine concordance with findings on TDCs. In Arm 2 and Arm 3, CRP of C-ETACs was performed using the CCAs indicated in SoC for the respective cancer type (Supplementary Table S4).

### Statistical analysis

In a prior retrospective analysis (unpublished data) of TDCs and C-TACs in cancer patients, it was observed that a 50% threshold for in vitro cell death had high concordance with clinical response/non-response to treatment. Hence, a median response / resistance threshold of 50% in vitro cell death at 12 h post-initiation of drug exposure was considered appropriate for response evaluation and used for purpose of correlation between C-TACs and TDCs. Samples which showed < 50% cell death were annotated as Resistant (‘R’) while those with ≥ 50% cell death were annotated as Sensitive (S). The Statistical R [15] v3.5.2 Package was used for all statistical analysis and graphical presentations. Pearson correlation coefficient was calculated correlation using cor (*x*, *y*) function in R.

## Results

### CCA sensitivities of C-TACs are concordant with TDCs

**Fig. 3:**
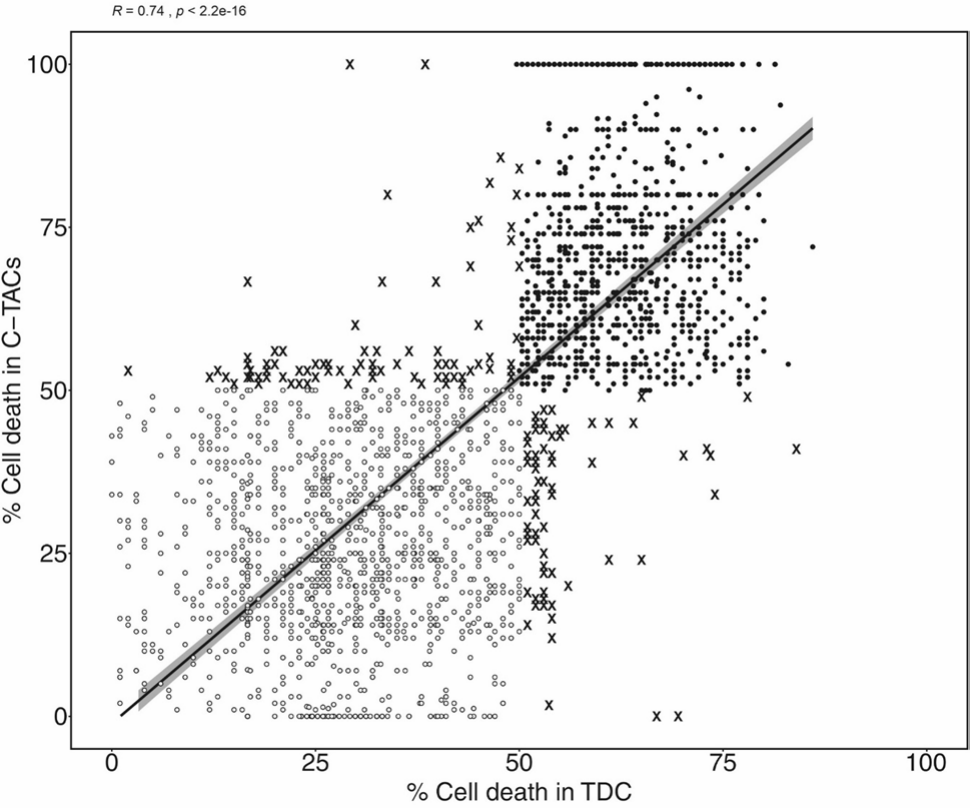
Chemoresistance concordance between C-TACs and TDCs. Correlation between cell-death (%) in C-TACs (Y-axis) and TDCs (X-axis) in each paired analysis (CTAC-drug-TDC). Clustering of data-points in the lower left and top right quadrants indicates high concordance between C-TACs and TDCs for Resistance (Open Circles) and Sensitivity (Closed Circles), respectively. Outliers are indicated as ‘X’. Linear regression and standard error are indicated

We determined whether the in vitro CRP of C-TACs and concurrent TDCs from the same patients were comparable. There were 2593 unique paired combinations of C-TAC: Drug: TDC from the 230 patients in Arm1. When the drug-response (resistance/sensitivity) of TDCs was mapped to the corresponding paired C-TACs there was concordance in 2428 (93.7%) combinations and discordance in 165 (6.3%) samples. High concordance was uniformly observed across all cancer types (Supplementary Table S5) and yielded an overall correlation coefficient *R* = 0.79 with *p* < 2.2 × 10^–16^ (Fig. 3).

### C-TACS inform de novo CCA sensitivity in treatment-naïve patients

**Fig. 4:**
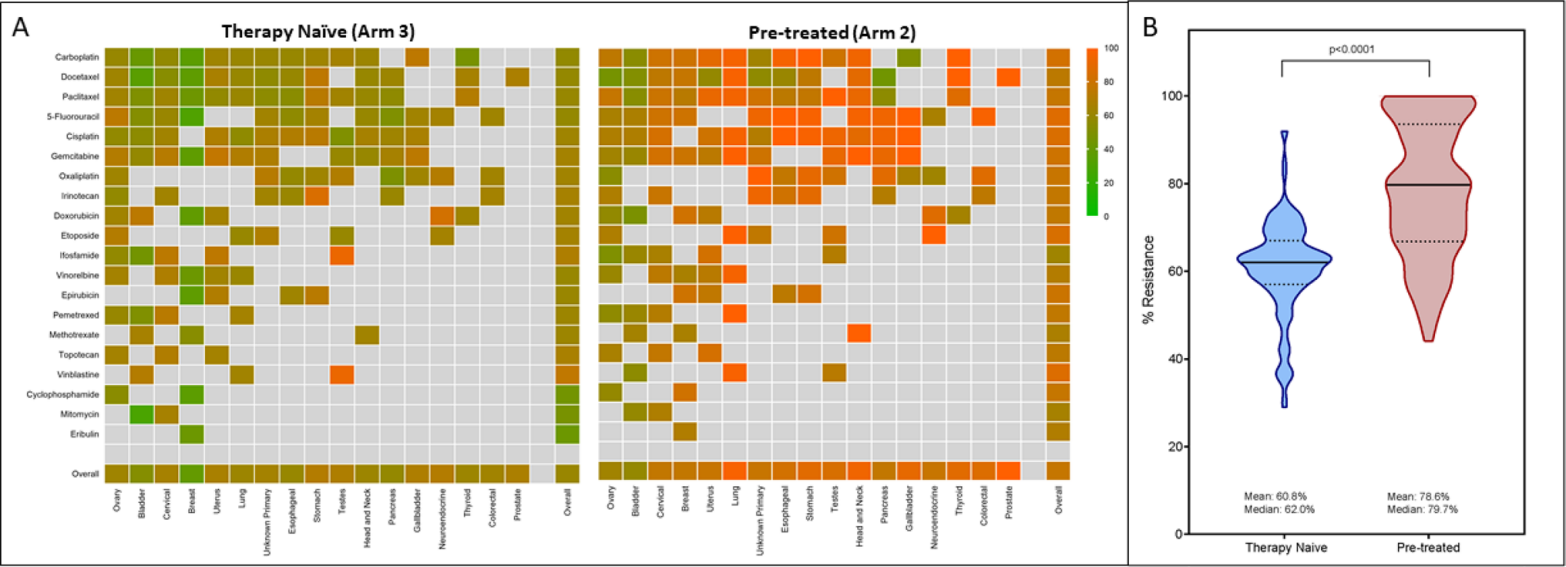
Innate and acquired chemoresistance. **a** Heat-map panels depicts incidence rate (%) of chemoresistance in C-TACs per cancer type and CCA in therapy naïve (left) and pretreated (right) sub-cohorts. Lowermost rows indicate cumulative heat per cancer type while rightmost columns indicate cumulative heat per CCA. Warmer shade indicates higher resistance. **b** Violin Plot depicting median and range of cumulative Innate and Acquired chemoresistance in C-TACs from Therapy Naïve and Pretreated patients

Among the cohort of 2734 therapy naïve patients in Arm 3, there were 37,542 unique C-TAC: CCA combinations. Drug resistance was observed in 22,109 (58.9%) combinations, which amounted to resistance towards ≥ 1 CCA in 1662 (60.8%) patients. This finding indicated the existence of C-TAC populations with intrinsic insensitivity to CCA. Cancer-wise innate chemo-resistance of C-TACs towards CCAs is depicted in Fig. 4a. In a subset of patients (*n* = 77) from this arm, radiological outcome data were obtained following administration of first-line regimens (non-surgical). The treating clinicians and the laboratory were both blinded to each other. Patients were followed-up after 6 months or completion of prerequisite cycles of therapy. Among 33 patients where C-TACs showed complete or predominant sensitivity to drugs in respective treatment regimens, 32 patients achieved CR or PR at a follow-up PET-CT scan indicating 97% in vitro*: *in vivo concordance. In the remaining 44 patients, C-TACs showed predominant lack of sensitivity to the drugs in the treatment regimens leading to absence of radiological response in 41% of patients (Supplementary Table S6).

### C-TACs accurately represent previous chemotherapy exposure

Among cohort of 2201 pretreated patients in Arm 2, there were 16,331 unique paired C-TAC: drug combinations in the context of the CCA history of each patient. Drug resistance was noted in 12,707 combinations (77.8%) amounting to resistance towards ≥ 1 CCA in 1730 (78.6%) patients. This finding suggests that in most cases C-TACs had acquired resistance to previously administered anticancer agents. Cancer-wise acquired chemotherapy resistance of C-TACs towards CCAs is depicted in Fig. 4b. C-TACs were obtained from a sub-group of patients for prospective comparison between in vitro CCA response and in vivo radiological status. Among 143 patients where the cancer showed radiological disease progression following administration of SoC CCA, C-TACs from 124 patients showed in vitro resistance to drugs in the administered treatments thus indicating 86.7% in vitro*: *in vivo concordance (Supplementary Table S6).

## Discussion

Though it is agreed that timely identification of drug resistance is critical for optimal therapy management, there are presently no technologies or biomarkers for real time surveillance or prospectively determining drug resistance. Upfront knowledge of innate drug resistance and early detection of emerging acquired resistance thus have major clinical and financial implications, especially if such knowledge can be obtained non-invasively and in real-time. There are a few commercial assays which examine the CRP of TDC, which, however, require a substantial amount of tissue from an invasive biopsy, have extended turn-around times, and have low or no correlation with clinical outcomes [16, 17]. CRP of TDCs is clinically unviable for two further reasons, (a) tumor evolution and heterogeneity render CRP from diagnostic biopsy rapidly obsolete with time and disease progression, and (b) repetitive invasive biopsies to obtain cells from tumor tissue are most often clinically unadvisable. Together, these factors have greatly restricted the adoption of these platforms into routine clinical practice.

It is well accepted that blood is a viable option for real-time sampling of tumor analytes. We hence describe the use of C-TACs for functional chemo-response profiling of cancers. C-TACs include CTCs (EpCAM + , PanCK + , CD45-) as well as CD45 + cells such as TAM and TAF that can be profiled to obtain clinically informative data. Prior reports have acknowledged the therapeutic potential of targeting tumor associated cells (such as TAM) owing to their role in suppressing antitumor immunity and promoting tumor progression [18]. The negative enrichment approach we developed [12] for harvesting of C-TACs using epigenetically activated media yielded consistently high numbers of viable C-TACs across all cancer types and permits meaningful evaluation of chemotherapy sensitivity/resistance using a broad panel of cytotoxic anticancer agents. Direct functional interrogation of viable C-TACs can provide actionable information, which is clearly more useful clinically than simple numerical or molecular correlation of such circulating malignant cells with disease status [5, 19]. Since the presence of viable tumor cells in peripheral blood has been causatively linked to metastasis, understanding their drug sensitivities may aid detection of emergent chemotherapy-resistant clonal sub-populations [20, 21].

The study findings demonstrate a robust correlation between CRP of C-TACs and TDCs implying that C-TACs accurately represent and report the chemotherapy sensitivity characteristics of the tumor from which they derive in the vast majority of cases.

In the arm of therapy naïve patients, C-TACs displayed widely variable innate resistance consistent with the clinical setting where lack of response to first line CCA is commonly encountered in multiple cancer types. For example, in metastatic breast cancer, response rates to first-line Taxol or Capecitabine are typically around 30% with stable disease achieved in a further 30%. With sub-optimal Pathological Complete Response (pCR) rates in the neo-adjuvant setting [22, 23]. Similar treatment failures have been reported following resistance to 5-Fluorouracil combination regimens (FOLFOX, FOLFIRI) have been reported in Colorectal cancers [24]. Likewise, resistance to first line platinum regimens are encountered in cancers of the Head and Neck, Oesophagus, Stomach, Colorectum, Ovary, Breast, Lung and Gallbladder [25]. Detection of chemo-resistant C-TACs in therapy naïve patient samples can be predictive of sub-optimal response as well as eventual disease progression, which is clearly advantageous prior to initiation of treatment. Similarly, CRP of C-TACs from previously treated patients detected higher chemoresistance in a majority of samples indicating acquired resistance following failure of/exposure to prior therapies. The ability to detect emergent (acquired) resistance indicates high accuracy for longitudinal monitoring where it would be possible to identify such ‘resistance-educated’ C-TACs. Additionally, it is also possible to identify agents from prior regimens that may be used to re-challenge the tumor in a subsequent line of therapy.

The clinical utility of CRP of CTACs was investigated in the real-world scenario by assessing concordance between in vitro findings and objective (radiological) measurement of treatment response. Patients who were therapy naïve at initial CRP were followed-up to determine response to first line treatments. Within this subgroup, we observed ~ 97% concordance between CR or PR and in vitro sensitivity of C-TACs to CCA. On the other hand, a lower in vitro sensitivity was associated with lower chance of radiological response to treatment. In the first line setting, in vitro sensitivity in CRP was thus more predictive of response to therapy. In the second subset-arm, we evaluated CRP in patients who were already receiving CCA prior to a follow-up radiological scan. Among the patients with radiologically evident PD, we observed ~ 87% concordance between treatment response/resistance and in vitro sensitivity/resistance of C-TACs to CCA. CRP of C-TACs can non-invasively determine failed treatments with high accuracy and can be used for longitudinal monitoring of patients. In this pretreated population, chemoresistant C-TACs were observed even in patients with radiologically evident partial response (PR) to treatment. Since PR, by definition, indicates slower or no response to treatment in a proportion of the tumor, it is likely that the resistant C-TACs emerged from the non- or weakly responding tumors, indicating the presence of a surviving resistant tumor cell population which could pose a risk of treatment failure and resurgence.

Significant inter-patient variability within all cancer types was observed which indicated the need and potential value of this approach prior to any line of therapy including neo-adjuvant. CRP can avoid several pitfalls of present treatment structures, especially in pretreated patients, following failure of multiple lines of multi-drug regimens by identifying and eliminating potentially sub-optimal drugs and reduce the risk of unnecessary toxicity arising from sub-optimal agents. In vitro chemotherapy sensitivity/resistance profiling of C-TACs is a non-invasive, uncomplicated, cost-effective process to determine cancer cell sensitivity to CCA in real time. CRP can be performed not only at diagnosis (prior to first line therapy selection), but also routinely during ongoing cancer treatment to achieve a previously unattainable level of synchronicity, precision and personalization. Therapy selection based on CRP of C-TACs may not only be able to reduce the risk of progression or recurrence due to treatment failures, but also the expenses of sub-optimal treatments as well as the accumulated drug toxicities associated with failed treatments. The ability to obtain treatment insight in real time and non-invasively has profound clinical significance. This approach is not only mature for adoption in clinical practice but also for improving efficiency of clinical trials aimed at expanding the scope of approved CCAs for use in additional cancers apart from those that are included in the labelled indication.

## Electronic supplementary material

Below is the link to the electronic supplementary material.

Supplementary file1 (DOCX 906 KB)

## Data Availability

Deidentified data may be made available by the authors upon reasonable request and may require the execution of appropriate non-disclosure agreements.

## Abbreviations

CRP: Chemoresistance profiling
CTCs: Circulating tumor cells
C-TACs: Circulating tumor-associated cells
TDCs: Tumor tissue-derived cells
PBMCs: Peripheral blood mononuclear cells
DCG: Datar Cancer Genetics
NABL: National Accreditation Board for Testing and Calibration Laboratories
ILAC: International Laboratory Accreditation Cooperation
TAM: Tumor-associated macrophages
TAFs: Tumor-associated fibroblasts EpCAM: Epithelial cell adhesion molecule
PE: Phycoerythrin
CK/pan-CK: Pan-Cytokeratins
FITC: Fluorescein isothiocyanate
DAPI: 4,6–Diaminodino–2–phenylindole
CD45: Leucocyte common antigen
ICS: Instrument Control Software
KU: Kinetic units
HNSCC: Head and neck squamous cell carcinomas
NCCN: National Comprehensive Cancer Network
CCA: Cytotoxic chemotherapy agent
SoC: Standard of Care
