# Supplementary material for: Clinical utility of circulating tumor-associated cells to predict and monitor chemo-response in solid tumors
Source: Cancer Chemother Pharmacol. 2020 Nov 10;87(2):197–205. doi: 10.1007/s00280-020-04189-8 (PMC7870597; doi:10.1007/s00280-020-04189-8)
Supplement: Supplementary file 1 — Supplementary file1 (DOCX 906 KB) [file 280_2020_4189_MOESM1_ESM.docx]

**TITLE**

Clinical Utility of Circulating Tumor Associated Cells to Predict and Monitor Chemo Response in Solid Tumors

**AUTHORS**

Timothy Crook^1^, Andrew Gaya^2^, Raymond Page^3^, Sewanti Limaye^4^, Anantbhushan Ranade^5^, Amit Bhatt^5^, Sanket Patil^6^, Prashant Kumar^7,8^, Darshana Patil^6^, Dadasaheb Akolkar^*6^

**AFFILIATIONS**

^1^Department of Oncology, Broomsfield Hospital, Chelmsford, UK.

^2^HCA Healthcare UK, London W1G 6AF, United Kingdom

^3^Worcester Polytechnic Institute, Worcester, USA

^4^Department of Medical Oncology, Kokilaben Dhirubai Ambani Hospital, Mumbai, India.

^5^Department of Medical Oncology, Avinash Cancer Clinic, Pune, India.

^6^Datar Cancer Genetics, Nasik, India.

^7^Institute of Bioinformatics, International Technology Park, Bangalore 560066, Karnataka, India.

^8^ Manipal Academy of Higher Education (MAHE), Manipal 576104, Karnataka, India.

***CORRESPONDING AUTHOR**

Dr. Dadasaheb Akolkar, Datar Cancer Genetics,

F-8 D-Road, Ambad, Nasik – 422010, India.

[dadasaheb.akolkar@datarpgx.com](mailto:dadasaheb.akolkar@datarpgx.com)

ORCID ID: 0000-0002-4434-4887

**SUPPLEMENTARY DATA**

**Supplementary Video S1.** **Chemoresistance Profiling**. Time-lapse video depicting response of C-TACs from a breast cancer patient when the C-TACs were treated with individual drugs for 12 h. The information panel on the left indicates drugs and observed cell-death (%), time as well as scale.

**Supplementary Figure S1. Immunocytochemistry Profiling of C-TACs.** C-TACs identified by staining with (A) DAPI, (B) EpCAM, (C) Pan-CK and (D) CD45. Panel (E) is the composite image.

**Supplementary Figure S2.** Representative images of C-TACs profiled by ICC for Organ and Subtype Specific (OSS) markers (Supplementary Table 2). (A) Breast, (B) Lung (AD), (C) Head and Neck, (D) Cervix, (E) Colorectum, (F) Stomach, (G) Oesophagus, (H) Ovary. (I) Pancreas, (J) Prostate, (K) Bladder, (L) Neuroendocrine. Each set of 3 panels includes DAPI, respective OSS marker and the composite image.

**Supplementary Table S1. Yields of C-TACs in various Cancers.** The yield of C-TACs was comparable between all cancer types and there were no significant differences between C-TAC yields in pre-treated and therapy naïve patients. Sufficient C-TACs were obtained from blood samples of all study participants for in vitro chemoresistance profiling.

| **Cancer Type** | **Yield of C-TACs (cells / 15 mL), Median (Range)** | |
| --- | --- | --- |
|  | **Treatment Naïve** | **Pre-treated** |
| Bladder | 2332 (814 – 3940) | 1372 (903 – 3594) |
| Breast | 1492 (288 – 3983) | 1380 (328 – 3976) |
| Cervix | 1389 (455 – 3963) | 1464 (382 – 3923) |
| Colorectum | 1516 (404 – 3850) | 1430 (478 – 3851) |
| Gallbladder | 1377 (796 – 3840) | 1426 (758 – 1824) |
| Head and Neck | 1535 (291 – 3918) | 1434 (292 – 3846) |
| Lung | 1690 (448 – 3915) | 1521 (390 – 3902) |
| Neuroendocrine | 1307 (309 – 1927) | 1676 (471 – 2577) |
| Oesophagus | 1727 (453 – 3922) | 1387 (494 – 3915) |
| Ovary | 1640 (759 – 3925) | 1451 (404 – 3647) |
| Pancreas | 1689 (771 – 3929) | 1346 (635 – 3217) |
| Prostate | 1879 (512 – 3852) | 912 (821 – 1250) |
| Stomach | 1550 (771 – 2349) | 1374 (735 – 3732) |
| Testes | 1554 (897 – 1882) | 1511 (842 – 3508) |
| Thyroid | 1651 (1174 – 3990) | 1336 (927 – 1847) |
| Unknown Primary | 1599 (717 – 3929) | 1277 (907 – 3148) |
| Uterus | 1688 (499 – 3825) | 1477 (486 – 3578) |
| **Overall** | **1560 (288 – 3990)** | **1419 (292 – 3976)** |

**Supplementary Table S2. Organ and Subtype-Specific Markers.** ICC profiling of C-TACs was performed to identify organ of origin based on expression of the following OSS markers.

| **Organ** | **Subtype** | **Marker** |
| --- | --- | --- |
| Bladder | Urothelial Carcinoma | Uroplakin II |
| Breast | Adenocarcinoma | GCDFP-15 / GATA-3 |
| Cervix | Squamous Cell Carcinoma | P63 |
| Colorectum | Adenocarcinoma | CK-20 / CDX-2 |
| Gallbladder | Adenocarcinoma | CA19-9 / AMACR |
| Head and Neck | Squamous Cell Carcinoma | P63 |
| Lung | Adenocarcinoma | Napsin-A / TTF-1 |
| (Any) | Neuroendocrine Carcinoma | Synaptophysin / Chromogranin |
| Oesophagus | Squamous Cell Carcinoma` | CK5/6 |
| Ovary | Adenocarcinoma | CA125 / WT-1 |
| Pancreas | Adenocarcinoma | CK19 / CA19-9 |
| Prostate | Adenocarcinoma | PSMA / AMACR |
| Stomach | Adenocarcinoma | CK-7 |
| Testes | Germ Cell Tumor | EpCAM / Pan-CK |
| Thyroid | Adenocarcinoma | TTF-1 |
| Unknown | - | EpCAM / Pan-CK |
| Uterus | - | EpCAM / Pan-CK |

**Supplementary Table S3.** CCAs reported in the present study and observed cytotoxicities in cell lines reported as Kinetic Units (% Cell Death).

| **Drug** | **Cytotoxicity in Cell Lines** | | | **Arm 1** | **Arm 2** | **Arm 3** |
| --- | --- | --- | --- | --- | --- | --- |
|  | **SKBR** | **RCA** | **SW620** |  |  |  |
| 5-Fluorouracil | 6.2 (58 %) | 3.5 (40 %) | 4.7 (48 %) | Y | Y | Y |
| Eribulin | 4.5 (46 %) | 2.9 (36 %) | 2.9 (36 %) | Y | Y | Y |
| Oxaliplatin | 3.4 (39 %) | 4.5 (46 %) | 5.1 (50 %) | Y | Y | Y |
| Pemetrexed | 4.8 (48 %) | 4.3 (45 %) | 5.2 (51 %) | Y | Y | Y |
| Methotrexate | 5.1 (50 %) | 4.8 (48 %) | 4.6 (47 %) | Y | Y | Y |
| Paclitaxel | 5.1 (50 %) | 5.7 (54 %) | 5.9 (56 %) | Y | Y | Y |
| Carboplatin | 4.9 (49 %) | 5.6 (54 %) | 3.7 (41 %) | Y | Y | Y |
| Cyclophosphamide | 4.5 (46 %) | 3.7 (41 %) | 4.2 (44 %) | Y | Y | Y |
| Cisplatin | 4.5 (46 %) | 5.6 (54 %) | 5.1 (50 %) | Y | Y | Y |
| Epirubicin | 3.8 (42 %) | 2.5 (33 %) | 3.4 (39 %) | Y | Y | Y |
| Gemcitabine | 4.5 (46 %) | 2.6 (34 %) | 3.6 (40 %) | Y | Y | Y |
| Topotecan | 4.5 (46 %) | 6.3 (58 %) | 3.2 (38 %) | Y | Y | Y |
| Docetaxel | 4.2 (44 %) | 3.8 (42 %) | 4.1 (44 %) | Y | Y | Y |
| Vinorelbine | 5.7 (54 %) | 5.4 (52 %) | 4.2 (44 %) | Y | Y | Y |
| Etoposide | 6.5 (60 %) | 7.7 (68 %) | 5.4 (52 %) | Y | Y | Y |
| Irinotecan | 4.5 (46 %) | 6.0 (56 %) | 4.1 (44 %) | Y | Y | Y |
| Doxorubicin | 5.3 (52 %) | 6.1 (57 %) | 5.7 (54 %) | Y | Y | Y |
| Mitomycin | 3.2 (38 %) | 4.1 (44 %) | 2.6 (34 %) | Y | Y | Y |
| Vinblastine | 5.2 (51 %) | 4.6 (47 %) | 3.4 (39 %) | Y | Y | Y |
| Ifosfamide | 3.6 (40 %) | 5.6 (54 %) | 4.2 (44 %) | Y | Y | Y |
| Cabazitaxel | 2.3 (32 %) | 3.1 (37 %) | 3.5 (40 %) | Y | - | - |
| Cytarabine | 2.6 (34 %) | 3.1 (37 %) | 3.4 (39 %) | Y | - | - |
| Temozolomide | 2.6 (34 %) | 3.5 (40 %) | 2.5 (33 %) | Y | - | - |
| Mitoxantrone | 2.4 (33 %) | 3.3 (38 %) | 4.1 (44 %) | Y | - | - |
| Daunorubicin | 4.0 (43 %) | 3.5 (40 %) | 5.1 (50 %) | Y | - | - |
| Dactinomycin | 5.1 (50 %) | 4.8 (48 %) | 3.8 (42 %) | Y | - | - |
| Vincristine | 3.4 (39 %) | 4.56 (47 %) | 4.9 (49 %) | Y | - | - |
| Bleomycin | 3.2 (38 %) | 4.9 (49 %) | 4.6 (47 %) | Y | - | - |
| Dacarbazine | 3.4 (39 %) | 2.5 (33 %) | 3.1 (37 %) | Y | - | - |
| Decitabine | 3.4 (39 %) | 3.5 (40 %) | 4.3 (45 %) | Y | - | - |
| Trabectedin | 2.9 (36 %) | 4.1 (44 %) | 4.3 (45 %) | Y | - | - |
| Procarbazine | 2.4 (33 %) | 2.4 (33 %) | 3.1 (37 %) | Y | - | - |

**Supplementary Table S4. Cancer-wise CCA Panel**

| **Drugs** | Bladder | Breast | Cervix | Colorectum | Oesophagus | Gallbladder | Head and Neck | Lung | Neuro-endocrine | Ovary | Pancreas | Prostate | Stomach | Testes | Thyroid | Unknown Primary | Uterus |
| --- | --- | --- | --- | --- | --- | --- | --- | --- | --- | --- | --- | --- | --- | --- | --- | --- | --- |
| 5-fluorouracil | Y | Y | Y | Y | Y | Y | Y | - | Y | Y | Y | - | Y | - | - | Y | - |
| Bleomycin | - | - | - | - | - | - | - | - | - | Y | - | - | - | Y | - | - | - |
| Carboplatin | Y | Y | Y | - | Y | Y | Y | Y | - | Y | - | - | Y | Y | Y | Y | Y |
| Cisplatin | Y | - | Y | - | Y | Y | Y | Y | - | Y | Y | - | Y | Y | - | Y | Y |
| Cyclophosphamide | - | Y | - | - | - | - | - | - | - | Y | - | - | - | - | - | - | - |
| Dacarbazine | - | - | - | - | - | - | - | - | Y | - | - | - | - | - | - | - | Y |
| Docetaxel | Y | Y | Y | - | Y | - | Y | Y | - | Y | Y | Y | Y | - | Y | Y | Y |
| Doxorubicin | Y | Y | - | - | - | - | - | - | Y | Y | - | - | - | - | Y | - | Y |
| Epirubicin | - | Y | - | - | Y | - | - | - | - | - | - | - | Y | - | - | - | Y |
| Eribulin | - | Y | - | - | - | - | - | - | - | - | - | - | - | - | - | - | - |
| Etoposide | - | - | - | - | - | - | - | Y | Y | Y | - | - | - | Y | - | Y | - |
| Gemcitabine | Y | Y | Y | - | - | Y | Y | Y | - | Y | Y | - | - | Y | - | Y | Y |
| Ifosfamide | Y | - | Y | - | - | - | - | - | - | Y | - | - | - | Y | - | - | Y |
| Irinotecan | - | - | Y | Y | Y | - | - | - | - | Y | Y | - | Y | - | - | Y | - |
| Methotrexate | Y | Y | - | - | - | - | Y | - | - | - | - | - | - | - | - | - | - |
| Mitomycin | Y | - | Y | - | - | - | - | - | - | - | - | - | - | - | - | - | - |
| Oxaliplatin | - | - | - | Y | Y | Y | - | - | Y | Y | Y | - | Y | Y | - | Y | - |
| Paclitaxel | Y | Y | Y | - | Y | - | Y | Y | - | Y | Y | - | Y | Y | Y | Y | Y |
| Pemetrexed | Y | - | Y | - | - | - | - | Y | - | Y | - | - | - | - | - | - | - |
| Temozolomide | - | - | - | - | - | - | - | - | Y | - | - | - | - | - | - | - | Y |
| Topotecan | - | - | Y | - | - | - | - | - | - | Y | - | - | - | - | - | - | Y |
| Vinblastine | Y | - | - | - | - | - | - | Y | - | - | - | - | - | Y | - | - | - |
| Vinorelbine | - | Y | Y | - | - | - | - | Y | - | Y | - | - | - | - | - | - | Y |

**Supplementary Table S5. Overall and cancer-wise concordance between chemoresistance profiles of TDCs and C-TACs.** Chemoresistance profiles of paired C-TACs (from pre-biopsy blood samples) and TDCs (from biopsied tumor tissue) was evaluated in 230 patients in Arm 1. There were 2593 unique paired combinations of CTAC-Drug-TDC. The number of such combinations as well as the proportion concordant for resistance or sensitivity are indicated in the table below. Overall as well as cancer-wise concordance is presented. Please also see Figure 3.

| **Cancer Type**  **(Number of Patients)** | **Unique Combinations**  **(TDC-Drug-CTAC)** | **Combinations and Concordance (Number and %)** | | | |
| --- | --- | --- | --- | --- | --- |
|  |  | **(A) Resistance** | **(B) Sensitivity** | **Overall (A+B)** | **None** |
| Breast (68) | 733 | 366 (49.9%) | 336 (45.9%) | 702 (95.8%) | 31 (4.2%) |
| Cervix (19) | 212 | 107 (50.5%) | 91 (42.9%) | 198 (93.4%) | 14 (6.6%) |
| Colorectum (20) | 209 | 117 (56.0%) | 80 (38.3%) | 197 (94.3%) | 12 (5.7%) |
| Gallbladder (2) | 19 | 9 (47.4%) | 8 (42.1%) | 17 (89.5%) | 2 (10.5%) |
| Head and Neck (71) | 913 | 552 (60.5%) | 290 (31.7%) | 842 (92.2%) | 71 (7.8%) |
| Lung (8) | 69 | 33 (47.8%) | 33 (47.8%) | 66 (95.6%) | 3 (4.4%) |
| Oesophagus (7) | 78 | 32 (41.0%) | 41 (52.6%) | 73 (93.6%) | 5 (6.4%) |
| Ovary (16) | 158 | 74 (46.8%) | 70 (44.3%) | 144 (91.1%) | 14 (8.9%) |
| Pancreas (3) | 24 | 11 (45.8%) | 13 (54.2%) | 24 (100.0%) | - |
| Prostate (5) | 41 | 20 (48.8%) | 18 (43.9%) | 38 (92.7%) | 3 &7.3%) |
| Stomach (1) | 25 | 22 (88.0%) | - | 22 (88.0%) | 3 (12.0%) |
| Thyroid (3) | 44 | 28 (63.6%) | 10 (22.7%) | 38 (86.3%) | 6 (13.7%) |
| Uterus (7) | 68 | 32 (47.1%) | 35 (51.5%) | 67 (98.5%) | 1 (1.5%) |
| **Overall (230)** | **2593** | **1403 (54.1%)** | **1025 (39.6%)** | **2428 (93.7%)** | **165 (6.4%)** |

**Supplementary Table S6. CRP Findings and Radiological Response to CCA.** Resistance score indicates the proportion of individual anticancer drugs in the monotherapy / multi-drug regimen towards which CTACs were resistance. In therapy naïve patients, lower resistance score is associated with a higher chance of treatment success, and a higher resistance score is associated with a lower chance of treatment success. In pretreated patients with refractory cancers, higher resistance score corelated with greater chance of therapy failure.

| **Resistance Score** | **Number of Patients Exhibiting Each Type of Response** | | | **Total** | **Concordance** | |
| --- | --- | --- | --- | --- | --- | --- |
|  | **CR / PR** | **SD** | **PD** |  |  |  |
| **(A) Patients at First Line of Therapy** | | | | | | |
| **0%** | 26 | 1 | 0 | **27** | **32 / 33 = 96.9%**  **(Response)** | |
| **33%** | 5 | 0 | 1 | **6** |  |  |
| **50%** | 8 | 1 | 4 | **13** | **26 / 44 = 59.0%**  **(Response)** | |
| **67%** | 6 | 0 | 0 | **6** |  |  |
| **100%** | 10 | 1 | 14 | **25** |  |  |
| **Overall** | **55** | **3** | **19** | **77** | **-** | **-** |
| **(B) Pretreated Patients (Refractory Cancers)** | | | | | | |
| **0%** | - | - | 10 | **10** | **-** | **-** |
| **33%** | - | - | 0 | **0** | **-** | **-** |
| **50%** | - | - | 9 | **9** | **-** | **-** |
| **67%** | - | - | 2 | **2** | **124 / 143 = 86.7%**  **(Failure)** | |
| **100%** | - | - | 122 | **122** |  |  |
| **Overall** | **-** | **-** | **143** | **143** | **-** | **-** |

**Supplementary Figure S1**

**
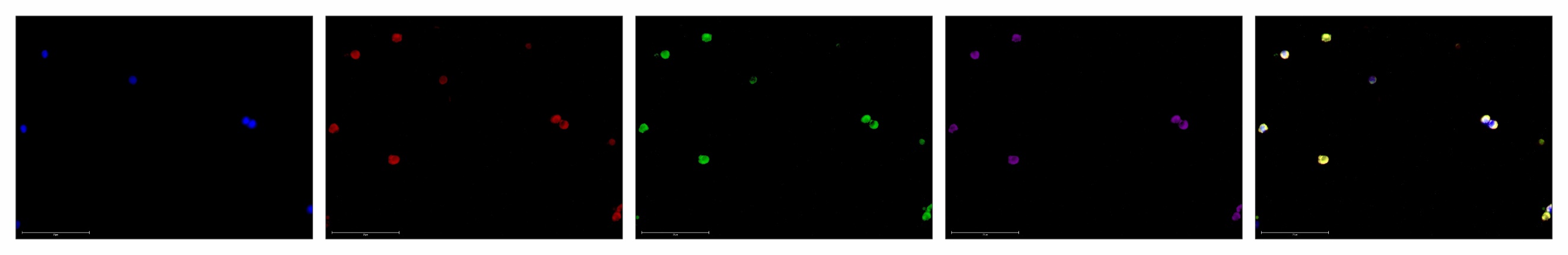
**

**Supplementary Figure S2**

**
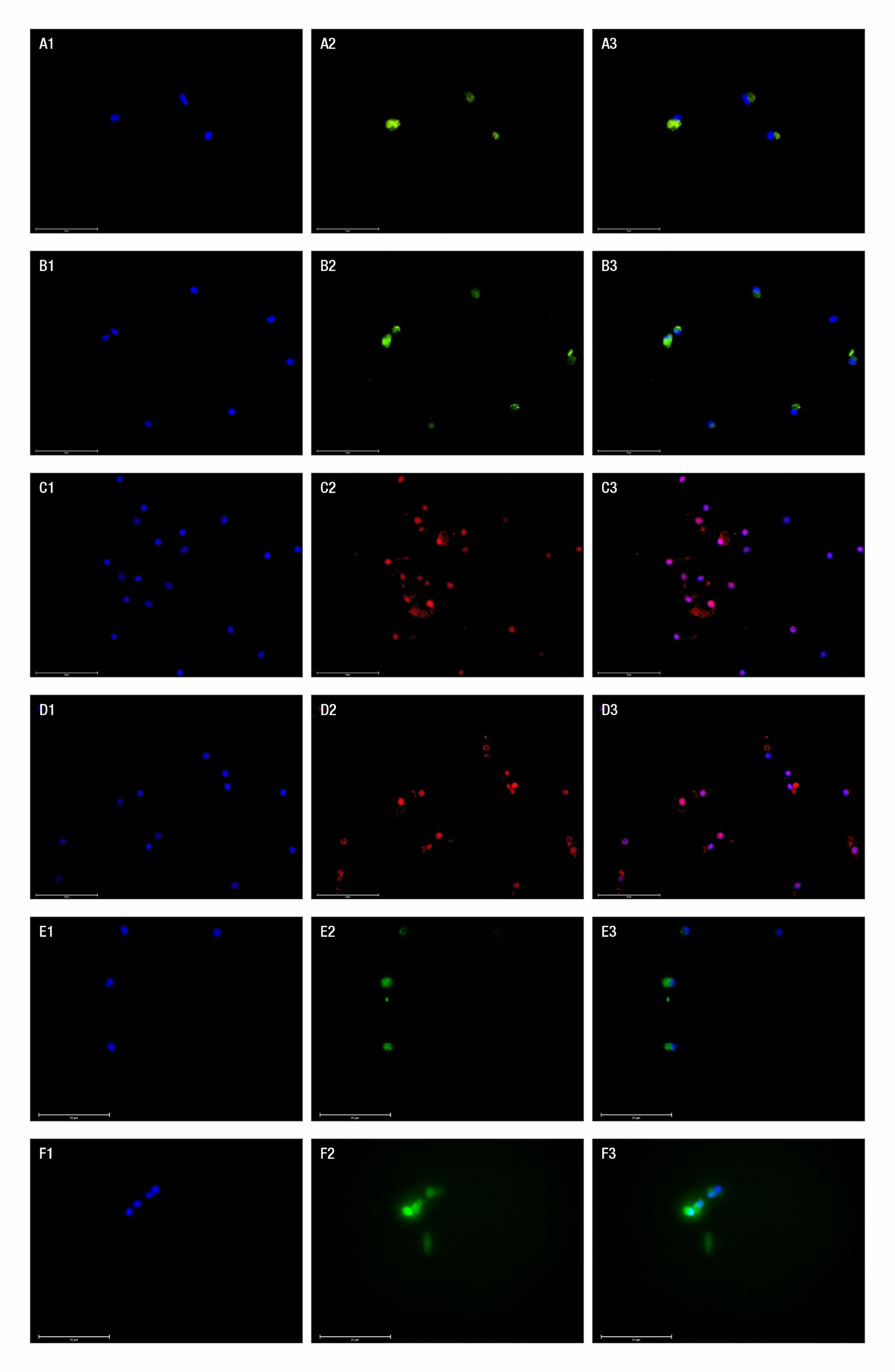
**

**
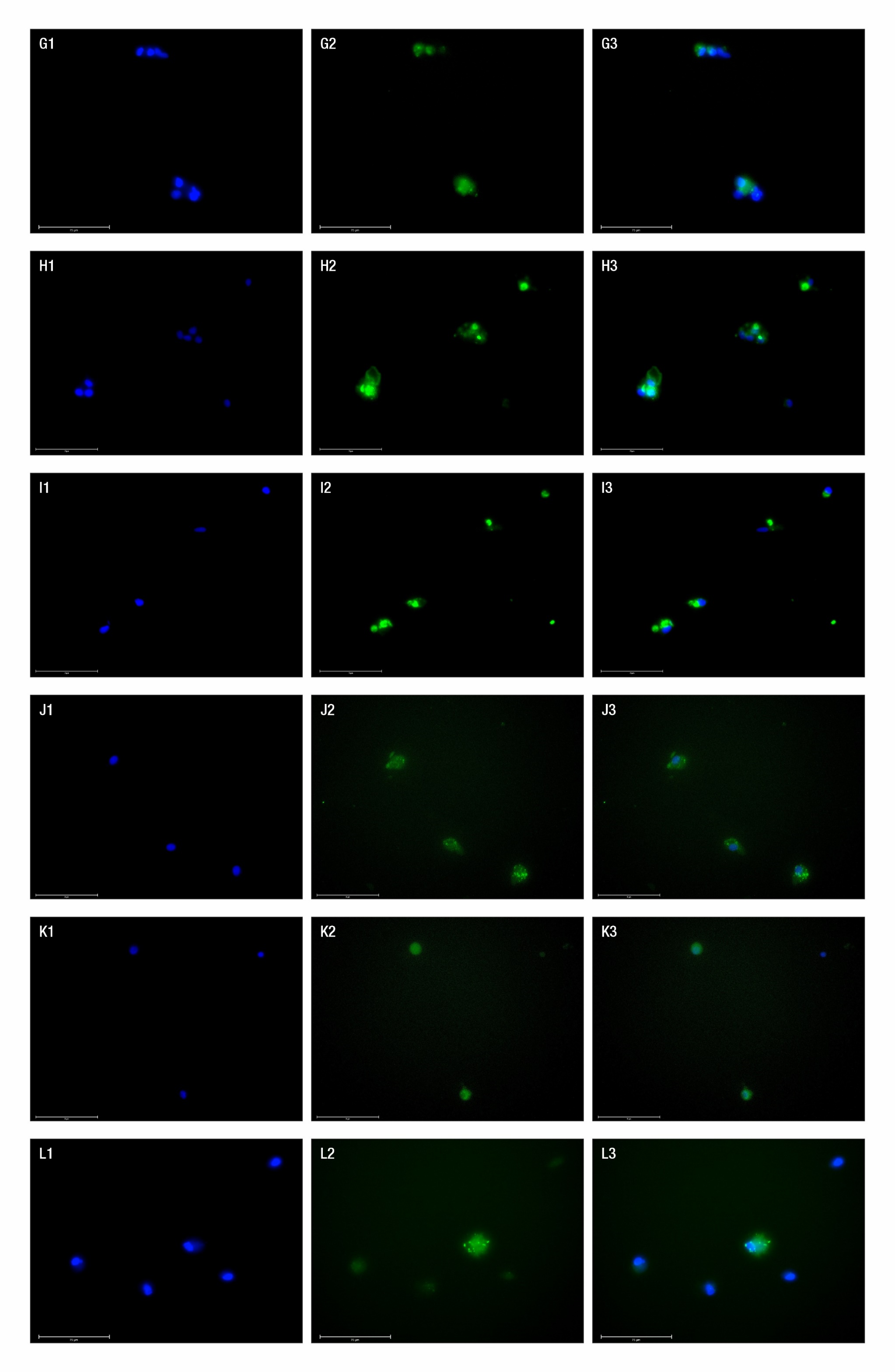
**
